# High prevalence of zoonotic trematodes in roach (*Rutilus rutilus*) in the Gulf of Finland

**DOI:** 10.1186/s13028-017-0343-7

**Published:** 2017-11-01

**Authors:** Anu Näreaho, Anna Maria Eriksson-Kallio, Petra Heikkinen, Anna Snellman, Antti Sukura, Perttu Koski

**Affiliations:** 1 Veterinary Biosciences, University of Helsinki, P.O. Box 66, 00014 Helsinki, Finland; 2 Finnish Food Safety Authority Evira, Mustialankatu 3, 00790 Helsinki, Finland; 3 Finnish Food Safety Authority Evira, Elektroniikkatie 3, 90590 Oulu, Finland

**Keywords:** *Holostephanus dubinini*, Metacercaria, *Metorchis bilis*, Posthodiplostomum, *Pseudamphistomum truncatum*, Roach, Trematode

## Abstract

The intention to increase roach (*Rutilus rutilus*) consumption is in focus for ecological and economic reasons in Finland. However, its safety as food has not been considered comprehensively. We collected and artificially digested 85 roach halves originating from the south-eastern coast of Finland, and found trematode metacercariae in 98.8% of the samples. Based on polymerase chain reaction (PCR) and sequencing of amplicons generated from the ITS2 gene region, zoonotic parasites of the family Opistorchiidae were identified as *Pseudamphistomum truncatum* and *Metorchis bilis*, and also non-zoonotic *Holostephanus dubinini* (family Cyathocotylidae) and *Posthodiplostomum* spp. (family Diplostomidae) were identified. The species identity of other trematodes found is currently being investigated. Mixed infections of several trematode species were common. The prevalence of morphologically identified zoonotic *P. truncatum* was 46%, and zoonotic *M. bilis* was found in one sequence sample. The high prevalence of zoonotic trematode metacercariae in roach from the Gulf of Finland is alarming. Only thoroughly cooked roach products can be recommended for human or animal consumption from the area.

## Findings

The roach (*Rutilus rutilus*) is in Finland considered as a coarse fish with low commercial value. In 2015, for example, the average annual consumption of roach in Finland was only 50 g per capita (counted as fillet weight) [1]. Its removal from the water system, however, reduces the biomass and delays eutrophication, and the utilization of roach as fertilizer, in bioenergy production, as animal feed, and recently as food, has consequently been under study [2]. Interest in ecological and local food has increased and the industrial use of roach in human nutrition has been tested in Finland. Roach patties have been produced in some industrial kitchens and served in work places, schools and nursing homes. The consumption of unprocessed, raw roach in Finland is still rare, but food trends including salt curing, raw pickling and sushi might change this.

Fishborne trematode infections are of major concern in areas of high prevalence with eating habits favouring the consumption of raw fish [3, 4]. Fishborne intestinal trematodiasis is common, for example, in certain parts of Asia, and a high prevalence has been reported from Vietnam [5]. Liver flukes may cause bile duct and liver damage and even bile duct cancer [4, 6, 7].

There have been previous observations of black spots on the skin and fins of roach caused by a bird trematode *Posthodiplostomum* (family Diplostomidae) in the brackish water of the Gulf of Finland, and their occurrence appears to be increasing [8]. Zoonotic *Pseudamphistomum truncatum* and *Metorchis bilis* (family Opistorchiidae) have been found in the coastal area in one of their final hosts, the fox [8]. Grey seals (*Halichoerus grypus*) in the Baltic Sea have also been observed to commonly carry *P. truncatum* [8, 9]. High prevalence (75%) of *Pseudamphistomum truncatum* in the roach in the Russian waters of the Gulf of Finland has been recently reported [10]. *Metorchis bilis*, nowadays genetically identified as a single species together with *M. albidus* and *M. crassiusculus* [11], as well as *P. truncatum*, can infect humans [12].

Due to infections in wildlife, we carried out a preliminary prevalence study on zoonotic trematodes in one of their intermediate hosts, the roach, from one location in the eastern Gulf of Finland, Baltic Sea. We also aimed to identify other trematode species existing in the area.

Roach caught by local commercial fishermen as by-catch were collected from the eastern Gulf of Finland, near the city of Kotka. They were transported to the Finnish Food Safety Authority (Evira) in Helsinki, where they were measured, weighed, gutted and filleted. Topical black spots were semi-quantified for each fish. Half of the fish, with the fins and skin included but without the head, was digested and the other half was frozen (− 20 °C) for further purposes. Altogether, 85 roach halves were digested and examined for metacercariae.

The digestion was performed in the parasite laboratory of the Faculty of Veterinary Medicine, University of Helsinki, with HCl-pepsin digestion modified from the method described by WHO [13]. Briefly, 50 g or smaller fish fillet was homogenized with a kitchen grinder and 500 mL of artificial gastric fluid containing 1% pepsin and 0.6% HCl was added. If the fish fillet was heavier, more digestion fluid was used accordingly. The mixture was placed on a magnetic stirrer and vigorously stirred for 30 min at 37 °C. The digestion fluid was then sieved through a kitchen sieve with a mesh size of about 2 mm into a funnel and allowed to sediment for 30 min. The sediment (about 1:5 of the original volume) was collected, mixed with tap water, sieved through a smaller mesh size (1 mm), and sedimented again for 15 min. This clarification step was repeated if the fluid was still too cloudy for microscopic examination. After the final sedimentation, the sediment was collected on a petri dish with a grid drawn on the bottom and examined under a stereomicroscope. Morphologically similar metacercariae from each sample were preserved in tubes in ethanol at − 20 °C for DNA analysis.

Molecular typing was performed for 83 individual metacercariae from 31 fish. Before lysis, the excess ethanol was evaporated from each sample tube containing a single metacercaria. Lysis buffer (10 mM Tris (pH 8.0), 1 mM EDTA, 0.45% (v/v) Tween 20 and 60 µg/mL of Proteinase K) was added and incubated at 65 °C for 3 h or until the parasite had completely degraded. Finally, the proteinase enzyme was inactivated at 95 °C for 10 min.

Molecular identification was based on polymerase chain reaction (PCR) and sequencing of amplicons generated from the ITS2 gene region using previously published primers (F: 5′-CTCGGCTCGTGTGTCGATGA-3′ and R: 5′-GCATGCARTTCAGCGGGTA-3′) [14]. PCR was carried out in a final volume of 20 μL containing 1 × DyNAzyme Buffer (Finnzymes, Vantaa, Finland), 0.25 mM dNTP (Finnzymes), 2 mM MgCl_2_, 1U DyNAzyme II DNA Polymerase (Finnzymes), 0.25 µM of each primer and 2 µL of the cell lysate. PCR was performed under the following conditions: initial denaturation at 95 °C for 5 min, 40 cycles at 94 °C for 30 s, 55 °C for 1 min and 72 °C for 1 min, followed by a final extension of 7 min at 72 °C. All PCR reactions were carried out in an XP Cycler (Bioer, Hangzhou, China). The PCR products were visualized in 1.5% agarose gel electrophoresis, excised, gel-purified using an E.Z.N.A.^®^ Gel Extraction Kit (Omega Bio-tek, Norcross, GA, USA) and sequenced using ABI technology (Applied Biosystems Co., Waltham, USA). Sequencing was performed using a BigDye Terminator v3.1 Cycle Sequencing Kit (Applied Biosystems Co.) The quality of the individual electropherograms was verified visually and sequences were analysed using MEGA 6 software [15].

**Fig. 1:**
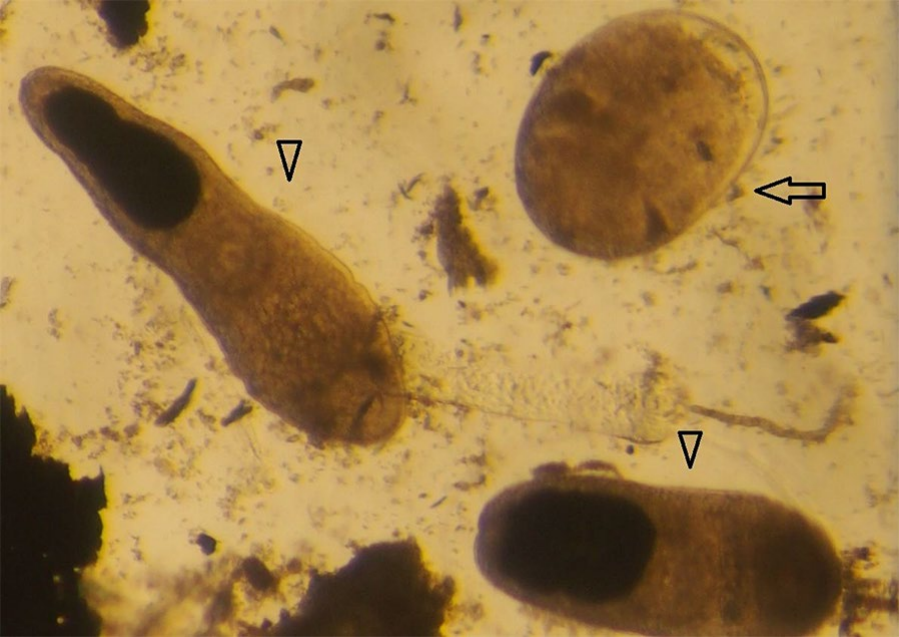
One encysted (arrow) and two excysted *Pseudamphistomum truncatum* metacercariae (arrowheads) in digestion fluid

All but one of the examined fish (98.8%) had trematode metacercariae in the digested half. Black spots, macroscopically typical of *Posthodiplostomum* species, were visually observed on 20% of the roach. The number of the metacercariae isolated from the digested halves varied from 0 to 281. The number of metacercariae per gram (mc/g) varied from 0 to 11.4 mc/g (median 0.32 mc/g). Mixed infections with several species were common. We observed 4 morphological categories of encysted metacercariae and 3 categories of excysted metacercariae, which overlapped in sequencing. From the sequenced metacercariae, 68 samples yielded a reliable sequence. Based on the sequencing, the trematode species in roach from the Gulf of Finland included at least zoonotic *P. truncatum* and *M. bilis,* and non-zoonotic bird trematodes, *Holostephanus dubinini* (family Cyathocotylidae) and *Posthodiplostomum* spp. (Table 1). In addition to these, currently unidentified species were present. *Pseudamphistomum truncatum* (Fig. 1) was also morphologically recognizable [16], and a total prevalence of 46% was calculated for this species by combining the morphological description and the sequence data from a total of 39 fish and by omitting any uncertain identifications.

**Table 1:** Length of the sequenced ITS2-region of the metacercariae and the correspondence to the GenBank data

Trematode	n sequences	Sequence length	Similarity %	Accession numbers
*P. truncatum*	23	388	100	JF710315
*M. bilis*	1	405	100	KT740982
*H. dubinini*	1	470	100	AY245707
*Posthodiplostomum* spp.	9	427	96	AB693170

A surprisingly high prevalence of trematode metacercariae was found in roach from the eastern Gulf of Finland. The abundance of the zoonotic species (mainly *Pseudamphistomum truncatum*) is alarming. Because the roach is considered as an ecologically recommendable food species [17], human consumption of raw roach should be carefully considered from the zoonotic point of view. Based on these preliminary results, only highly processed roach products with no possibility of containing infective trematode metacercariae can be recommended for human consumption or as animal feed. To date, no surveys on risk populations have been carried out, and no data are available on human or companion animal exposure. Until further investigations are carried out, all raw roach should be considered as risk material for humans and fish-eating pets, such as dogs and cats. In Ireland *P. truncatum* has been found in mink and Eurasian otter [18], and in Denmark in both roach and mink close to Copenhagen city centre, where concern over human health was also raised [16].

Future research in Finland should include: (1) species identification of all the roach trematodes found, (2) identification of their local definitive hosts and determining the prevalence in the first intermediate hosts (gastropods), which maintain the life cycle of the parasites, (3) evaluation of the health risks to humans and pets in the highly endemic area and (4) a wider investigation of the distribution of trematodes in the Baltic Sea coast as well as in the Finnish lakes and rivers.

## Abbreviations

dNTP: deoxynucleotide triphosphate
HCl: hydrochloric acid
ITS: internal transcribed spacer
mc/g: metacercariae per gram
MgCl2: magnesium chloride
PCR: polymerase chain reaction
